# In Vitro and In Silico Anti-Glioblastoma Activity of Hydroalcoholic Extracts of *Artemisia annua* L. and *Artemisia vulgaris* L.

**DOI:** 10.3390/molecules29112460

**Published:** 2024-05-23

**Authors:** Jurga Bernatoniene, Emilija Nemickaite, Daiva Majiene, Mindaugas Marksa, Dalia M. Kopustinskiene

**Affiliations:** 1 Department of Drug Technology and Social Pharmacy, Faculty of Pharmacy, Medical Academy, Lithuanian University of Health Sciences, Sukileliu pr. 13, LT-50161 Kaunas, Lithuania; jurga.bernatoniene@lsmuni.lt (J.B.); emilija.nemickaite@stud.lsmu.lt (E.N.); daiva.majiene@lsmuni.lt (D.M.); 2 Institute of Pharmaceutical Technologies, Faculty of Pharmacy, Medical Academy, Lithuanian University of Health Sciences, Sukileliu pr. 13, LT-50161 Kaunas, Lithuania; 3 Laboratory of Biochemistry, Neuroscience Institute, Lithuanian University of Health Sciences, Eiveniu Street 4, LT-50162 Kaunas, Lithuania; 4 Department of Analytical and Toxicological Chemistry, Medical Academy, Lithuanian University of Health Sciences, LT-50161 Kaunas, Lithuania; mindaugas.marksa@lsmu.lt

**Keywords:** glioblastoma, C6 glioma cell line, *Artemisia annua* L., *Artemisia vulgaris* L., chlorogenic acid

## Abstract

Glioblastoma, the most aggressive and challenging brain tumor, is a key focus in neuro-oncology due to its rapid growth and poor prognosis. The C6 glioma cell line is often used as a glioblastoma model due to its close simulation of human glioma characteristics, including rapid expansion and invasiveness. Alongside, herbal medicine, particularly Artemisia spp., is gaining attention for its anticancer potential, offering mechanisms like apoptosis induction, cell cycle arrest, and the inhibition of angiogenesis. In this study, we optimized extraction conditions of polyphenols from *Artemisia annua* L. and *Artemisia vulgaris* L. herbs and investigated their anticancer effects in silico and in vitro. Molecular docking of the main phenolic compounds of *A. annua* and *A. vulgaris* and potential target proteins, including programmed cell death (apoptosis) pathway proteins proapoptotic Bax (PDB ID 6EB6), anti-apoptotic Bcl-2 (PDB ID G5M), and the necroptosis pathway protein (PDB ID 7MON), mixed lineage kinase domain-like protein (MLKL), in complex with receptor-interacting serine/threonine-protein kinase 3 (RIPK3), revealed the high probability of their interactions, highlighting the possible influence of chlorogenic acid in modulating necroptosis processes. The cell viability of rat C6 glioma cell line was assessed using a nuclear fluorescent double-staining assay with Hoechst 33342 and propidium iodide. The extracts from *A. annua* and *A. vulgaris* have demonstrated anticancer activity in the glioblastoma model, with the synergistic effects of their combined compounds surpassing the efficacy of any single compound. Our results suggest the potential of these extracts as a basis for developing more effective glioblastoma treatments, emphasizing the importance of further research into their mechanisms of action and therapeutic applications.

## 1. Introduction

Glioblastoma, a very aggressive brain tumor, is a primary area of interest in the field of neuro-oncology because of its fast proliferation and unfavorable prognosis [1,2]. This malignant tumor originates in the glial cells of the brain, which provide support and insulation between neurons [1,2]. Due to its highly invasive nature, glioblastoma not only proliferates at an alarming rate but also infiltrates surrounding brain tissue, making surgical removal extremely difficult and often incomplete. The average survival times of patients diagnosed with glioblastoma typically does not exceed 15–18 months [1,2,3].

The standard treatment regimen of glioblastoma includes surgical resection followed by radiation and chemotherapy [3,4,5]. However, the effectiveness of these treatments is limited due to the resilience of the tumor and its ability to develop resistance to therapy [1,2,3,4]. The aggressive characteristics of glioblastoma highlight the critical need for innovative and diverse treatment strategies, such as herbal therapies [5,6,7]. In neuro-oncology, there is a growing interest in investigating the biochemical mechanisms through which herbal compounds interact with glioblastoma cells, to check on how these natural substances can inhibit tumor growth, disrupt the metabolic pathways essential for tumor survival, and induce apoptosis in cancerous cells [6,7,8]. The pharmacological effects of plant-based compounds on glioblastoma might lead to novel alternative treatments that are not only effective but also potentially less toxic than conventional chemotherapy.

**Figure 1:**
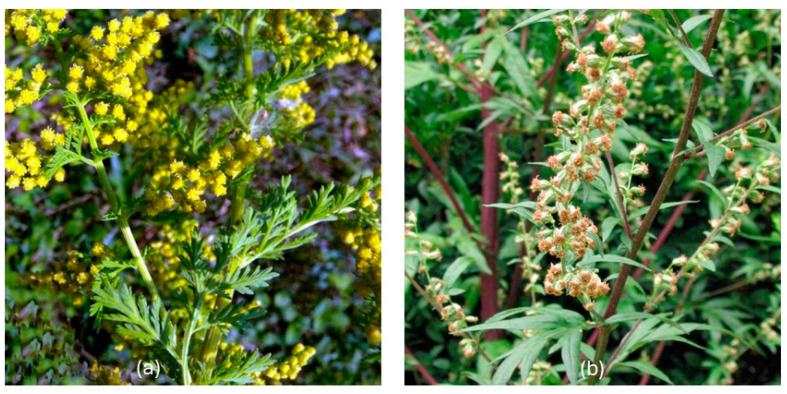
Plants of *Artemisia annua* L. (**a**) and *Artemisia vulgaris* L. (**b**).

*Artemisia annua* L. (Figure 1a), also known as sweet wormwood or annual wormwood, has shown potential anti-malarial, antimicrobial, antiviral, anti-inflammatory, and antioxidant properties; also, its anticancer properties are currently under extensive investigation. Traditionally, in Chinese medicine, it has been used for centuries to treat fever and other diseases and is included in various herbal formulations [9,10,11,12]. *Artemisia vulgaris* L. (Figure 1b, commonly known as mugwort, is used in alternative medicine for digestive issues, irregular menstrual cycles, high blood pressure, insomnia, epilepsy, and as a liver tonic and mild laxative. Prior to the introduction of hops, leaves and roots of *A. vulgaris* were used in cooking and brewing beer due to its aromatic properties [13,14,15].

The chemical composition of the *Artemisia* species is complex and includes a wide range of bioactive compounds [12,16]. The most notable component is artemisinin, a sesquiterpene lactone with a unique peroxide bridge, primarily responsible for its potent antimalarial activity. *A. annua* and *A. vulgaris* contain other sesquiterpenes, enhancing their therapeutic effects [12,16]. Flavonoids in *A. annua* and *vulgaris*, such as flavones (apigenin, luteolin, acacetin, etc.) and flavonols (artemetin, eupatin, rutin, etc.), are known for their antioxidant properties, and contribute to their anti-inflammatory and potential anticancer activities. These plants produce various essential oils, including camphor, borneol, cineole, and pinene, which account for their aromatic properties and may have therapeutic benefits. Phenolic acids like caffeic and chlorogenic acid in these plants are recognized for their antioxidant properties. Coumarins, alkaloids, terpenoids, and sterols further contribute to their medicinal value. Moreover, *A. annua* and *A. vulgaris* encompass a range of vitamins, minerals, and amino acids, enhancing their overall health benefits [12,14,16]. In recent reviews, *A. vulgaris* was reported to contain a variety of secondary metabolites, including the following numerous flavonoids isolated from the whole plant: flavones such as tricine, jaceosidin, eupafolin, chrysoeriol, diosmetin, apigenin, and luteolin; flavone glycosides like luteolin 7-glucoside and vitexin; flavanones such as homoeriodictyol and eriodictyol; flavonols like isorhamnetin; and flavonol glycosides including kaempferol and quercetin derivatives. Eriodictyol and luteolin were identified as the most abundant of these compounds [16,17]. It is important to note that, while artemisinin is the most well-known and studied compound in *A. annua*, the synergistic effect of the various constituents may contribute to the overall therapeutic potential of *Artemisia* plants [12,14,16].

Phenolic compounds currently under extensive investigation for glioblastoma treatment include curcumin, resveratrol, and the phenolic components found in green tea such as epigallocatechin gallate [6,7,18]. Curcumin, derived from the turmeric plant, has been studied for its ability to inhibit tumor growth and induce apoptosis in glioblastoma cells. Its ability to cross the blood–brain barrier and affinity for lipids make it a promising central nervous system cancer treatment [18,19]. Resveratrol, a compound found in grapes and berries, is noted for its potential to inhibit the proliferation of glioblastoma cells and enhance the sensitivity of these cells to chemotherapy [8,18]. Epigallocatechin gallate has shown promise in inhibiting the invasive properties of glioblastoma cells and suppressing angiogenesis, which is critical for tumor growth and metastasis [20,21]. These examples highlight the therapeutic potential of phenolic compounds in targeting the complex biology of glioblastoma, making them a focal point for ongoing research and development in cancer treatment strategies [18].

Bioactive compounds from Artemisia plants, like artemisinin, have shown anticancer potential in preclinical research [22,23]. The artemisinin derivative artesunate has shown potent anticancer actions on glioblastoma cells in vitro, inhibiting angiogenesis, suppressing cell proliferation, and activating apoptosis [24]. A bioactive compound from Artemisia spp. plants, jaceosidin, could inhibit glioblastoma cell invasion and selectively change many signaling pathways associated with the proliferation and spread of cancer cells [25]. Therefore, the aim of this study was to optimize the extraction conditions for the maximum yield of bioactive phenolic compounds from *A. annua* and *A. vulgaris* and to investigate their anticancer effects in silico and in a glioblastoma model in vitro.

## 2. Results

The aim of this study was to optimize the extraction of the main phenolic compounds from *A. annua* and *A. vulgaris* and to test their anticancer activity in silico and in vitro using the C6 glioma cell line.

### 2.1. Quantification of Herbal Hydroalcoholic Extracts of A. annua and A. vulgaris

Diverse hydroalcoholic extracts of Artemisia spp. were formulated at concentrations of 100 mg/mL, 300 mg/mL, and 500 mg/mL, where milled botanical specimens were homogenized with ethanol in volumetric ratios of 50%, 60%, 70%, 80%, and 96% (*w*/*w*). After the *A. annua* and *A. vulgaris* hydroalcoholic extracts were made, they were analyzed using high-performance liquid chromatography (HPLC). The maximum extraction yield of polyphenols was obtained using a concentration of 500 mg/mL with 80% ethanol, which was subsequently selected for further studies.

**Figure 2:**
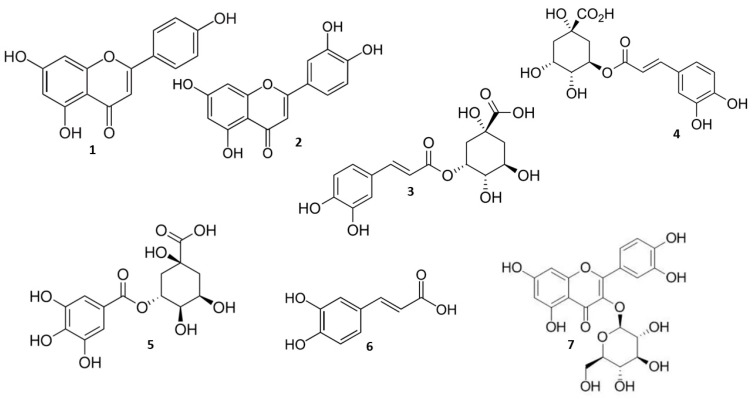
Chemical structures of main phenolic compounds detected in *Artemisia annua* L. and *Artemisia vulgaris* L. herbal hydroalcoholic extracts. **1**—apigenin, **2**—luteolin, **3**—neochlorogenic acid, **4**—chlorogenic acid, **5**—4-o-caffeoylquinic acid, **6**—caffeic acid, and **7**—isoquercitrin.

Seven polyphenolic compounds, namely apigenin, luteolin, neochlorogenic acid, chlorogenic acid, 4-o-caffeoylquinic acid, caffeic acid, and isoquercitrin (Figure 2), were identified through HPLC analysis of the samples of *A. annua* and *A. vulgaris* hydroalcoholic extracts in our study.

**Figure 3:**
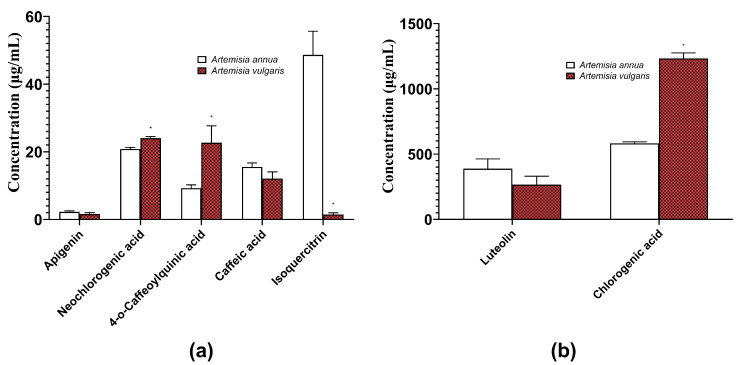
Yields of main polyphenolic compounds (low values (**a**), high values (**b**)) from herbal hydroalcoholic extracts of *Artemisia annua* L. and *Artemisia vulgaris* L. Data are presented as mean ± standard error (SEM), n = 4. * *p* < 0.05—statistically significant difference compared to corresponding *Artemisia annua* samples. The results were analyzed with a one-way analysis of variance (ANOVA) followed by Tukey’s multiple comparison test.

The total amount of phenolic compounds was 1066.28 μg/mL in the *A. annua* extract and 1560.58 μg/mL in the *A. vulgaris* extract. The most abundant polyphenol in both *A. annua* and *A. vulgaris* extracts was chlorogenic acid, followed by luteolin (Figure 3).

### 2.2. The Role of Excipients in the Optimization of Extraction Conditions of Bioactive Phenolic Compounds from Herbal Hydroalcoholic Extracts of A. annua and A. vulgaris

Excipients are essential for the optimization of extraction conditions of polyphenolic compounds, enhancing the efficiency and selectivity of the extraction process [26,27]. 

In our study, the extracts were enriched with titanium dioxide (2%), L-glutathione (1%), propylene glycol (10%), and β-cyclodextrin (5%); the results are summarized in Table 1.

Our results have shown that excipients titanium dioxide (2%) and L-glutathione (1%) significantly increased the yields of bioactive polyphenols; however, β-cyclodextrin (5%) was less effective in improving the extraction conditions (Table 1).

**Figure 4:**
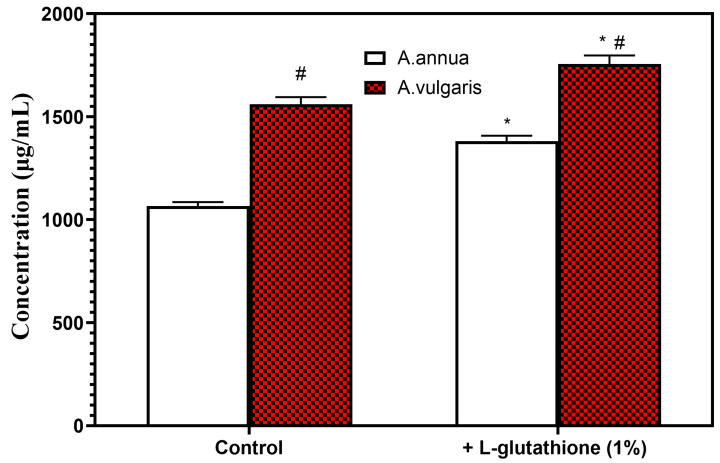
Total amount of phenolic compounds in herbal hydroalcoholic extracts of *Artemisia annua* L. and *Artemisia vulgaris* L. in the presence and absence of the excipient L-glutathione (1%). Data are presented as mean ± SEM, n = 4. * *p* < 0.05—statistically significant difference compared to control without the excipient; # *p* < 0.05—statistically significant difference of *A. vulgaris* samples compared to *A. annua* samples. The results were analyzed with a one-way analysis of variance (ANOVA) followed by Tukey’s multiple comparison test.

Since the European Food Safety Authority (EFSA) recommended phasing out the use of titanium dioxide due to its carcinogenic potential [28], we used L-glutathione (1%) as an excipient for further experiments (Figure 4).

The total amount of phenolic compounds with excipient L-glutathione (1%) increased compared to the control, both in the extracts of *A. annua* and *A. vulgaris* (Figure 4). Furthermore, more phenolic compounds were extracted from *A. vulgaris* than from *A. annua* (Figure 4).

### 2.3. In Silico Studies of Anticancer Activity of Main Bioactive Phenolic Compounds from Herbal Hydroalcoholic Extracts of Artemisia annua L. and Artemisia vulgaris L.

We conducted in silico studies of the main phenolic compounds of *A. annua* and *A. vulgaris* to identify potential lead compounds for additional studies on anticancer activity.

Programmed cell death (apoptosis) pathway proteins—proapoptotic Bax (PDB ID 6EB6), anti-apoptotic Bcl-2 (PDB ID 1G5M), and the necroptosis pathway protein (PDB ID 7MON), mixed lineage kinase domain-like protein (MLKL), in complex with receptor-interacting serine/threonine-protein kinase 3 (RIPK3)—have been chosen as potential target proteins.

**Figure 5:**
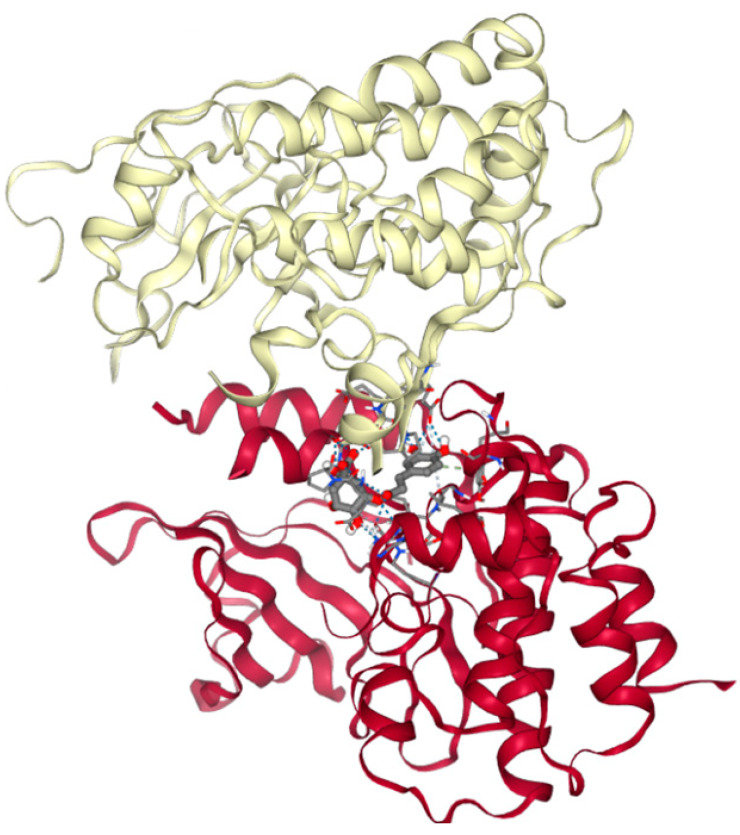
Docking of chlorogenic acid to MLKL/RIPK3 protein complex forms a necroptosis pathway. MLKL depicted in yellow, RIPK3 is depicted in red. Molecular docking studies were carried out using AutoDock Vina 4.05. All non-protein residues were removed, retaining pure protein structure for docking simulations. The structure presented has the lowest docking energy (−6.8 kcal/mol) and the highest number of hydrogen bonds (N18).

Molecular docking results are summarized in Table 2. Our results have shown that all the tested polyphenolic compounds from *A. annua* and *A. vulgaris* could dock to target proteins. The highest number of hydrogen bonds could be formed between chlorogenic acid and the MLKL/RIPK3 protein complex (Table 2, Figure 5), indicating the high probability of the possible interaction between them.

**Figure 6:**
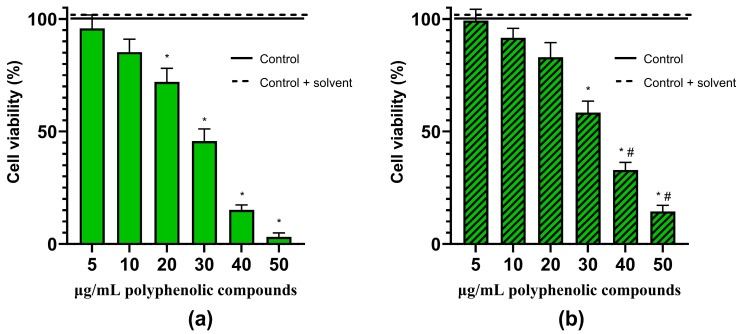
Effects of different concentrations of *Artemisia annua* L. extract without (**a**) and with the excipient—1% of L-glutathione (**b**) on the viability of C6 cells. C6 cells were treated with different concentrations of extract (5–50 µg/mL of phenolic compounds) for 24 h. Data are presented as means of percentage of the untreated control cells ± SEM (n = 5). * *p* < 0.05 versus control, ^#^ *p* < 0.05 versus extract without the excipient. The results were analyzed with one-way analysis of variance (ANOVA) followed by Tukey’s multiple comparison test.

Chlorogenic acid could form multiple hydrogen bonds between its oxygen atoms and Ser 373 and Arg 333 from the MLKL protein, as well as Ser 176, Cys 177, Glu 221, and Pro 223 from the RIPK3 protein (Figure 6). Also, it could form weak hydrogen bonds between its C5, C14, C16, and Ser 373 (MLKL), Glu 221 (RIPK3), and Gly 330 (MLKL), as well as pi stacking between its C16 and Tyr 389 (MLKL).

Since in silico studies have revealed the possibility of chlorogenic acid interactions with MLKL/RIPK3 necroptosis pathway proteins, and chlorogenic acid was the most abundant polyphenol of both *A. annua* and *A. vulgaris* extracts, we have tested its anticancer activity in the next series of in vitro experiments.

### 2.4. Anticancer Activity of Herbal Hydroalcoholic Extracts of Artemisia annua L. and Artemisia vulgaris L. and its Main Bioactive Phenolic Compound—Chlorogenic Acid

**Figure 7:**
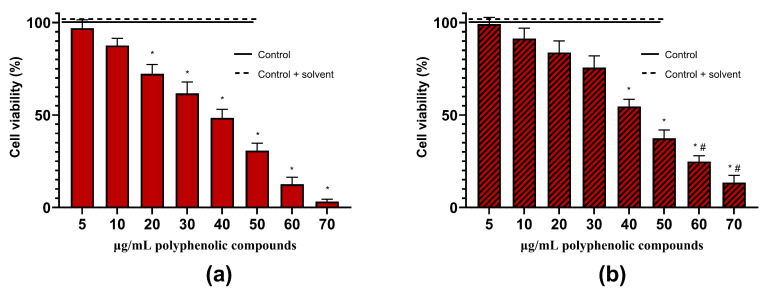
Effects of different concentrations of *Artemisia annua* L. extract without (**a**) and with the excipient—1% of L-glutathione (**b**) on viability of C6 cells. C6 cells were treated with different concentrations of extract (5–70 µg/mL of phenolic compounds) for 24 h. Data are presented as means of percentage of the untreated control cells ± SE (n = 5). * *p* < 0.05 versus control, ^#^ *p* < 0.05 versus extract without the excipient. The results were analyzed with one-way analysis of variance (ANOVA) followed by Tukey’s multiple comparison test.

**Figure 8:**
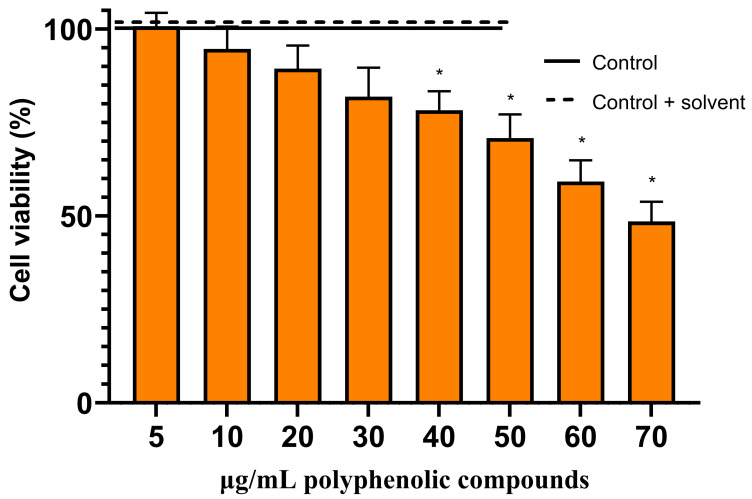
Effects of different concentrations of chlorogenic acid on viability of C6 cells. C6 cells were treated with different concentrations (5–70 µg/mL) of chlorogenic acid for 24 h. Data are presented as means of percentage of the untreated control cells ± SE (n = 5). * *p* < 0.05 versus control. The results were analyzed with one-way analysis of variance (ANOVA) followed by Tukey’s multiple comparison test.

We have evaluated the effects of hydroalcoholic *A. annua* (Figure 6) and *A. vulgaris* (Figure 7) extracts, as well as its main phenolic compound—chlorogenic acid—on the viability of glioblastoma C6 cells (Figure 8). For the control, the maximal concentrations of extracts used (70 µg/mL of phenolic compounds) or chlorogenic acid (70 µg/mL) were tested on the primary rat cerebellar neuronal-glial cell culture, neither of which was found to be cytotoxic. Also, the solvent ethanol had no effect on cell viability.

The lowest concentrations of extracts (5–10 µg/mL of phenolic compounds) had no significant effects on the cell viability (Figure 6 and Figure 7). *A. annua* (Figure 6) and *A. vulgaris* (Figure 7) extracts, at concentrations of 20 µg/mL of phenolic compounds, started to significantly decrease the viability of glioblastoma C6 cells, specifically, at a concentration of 30–40 µg/mL, by 41–84%.

The presence of L-glutathione in the samples had a tendency to preserve cells, probably due to its antioxidant activity. Cell viability was significantly higher by 10–18% in the samples treated with *A. annua* and *A. vulgaris* extracts with L-glutathione at concentrations of 40–70 µg/mL phenolic compounds, compared to the extracts without it (Figure 6 and Figure 7).

The main phenolic compound of *A. annua* and *A. vulgaris* extracts—chlorogenic acid—alone had lower activity than the extracts, starting to significantly decrease the viability of glioblastoma C6 cells at concentrations of 40–60 µg/mL of phenolic compounds (Figure 8). Chlorogenic acid at a concentration of 70 µg/mL of phenolic compounds decreased the viability of glioblastoma C6 cells by 48–52% (Figure 8).

**Figure 9:**
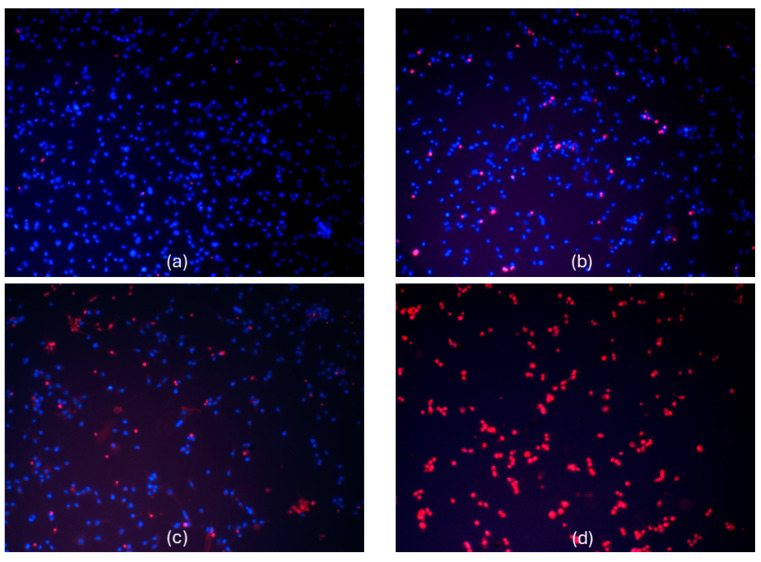
Effects of different concentrations of *A. annua* L. hydroalcoholic extract on viability of C6 cells. Cells were double-stained with Hoechst 33342 and PI, and the viability was assessed under fluorescence microscope. Original magnification ×20. Typical photographs of control cells (**a**) and after treatment with (**b**) 10 µg/mL phenolic compounds, (**c**) 20 µg/mL phenolic compounds (**d**) 50 µg/mL phenolic compounds of investigated extract. Hoechst 33342-positive cells, exhibiting blue fluorescence, were considered viable cells. PI-stained cells exhibiting red fluorescence were considered necrotic.

*A. annua* extract, at a concentration of 50 µg/mL of phenolic compounds, and *A. vulgaris* extract, at a concentration of 70 µg/mL of phenolic compounds, promoted necrotic death of all glioblastoma C6 cells in the samples (Figure 9).

Thus, our results demonstrate that *A. annua* and *A. vulgaris* extracts could exert anticancer activity in rat C6 glioma cells.

## 3. Discussion

### 3.1. Optimization of Extraction Conditions of Phenolic Compounds from Herbal Hydroalcoholic Extracts of Artemisia annua L. and Artemisia vulgaris L.

In our study, first we focused on optimizing the extraction conditions of *A. annua* and *A. vulgaris* to enhance the yield of bioactive polyphenols. In the extracts of both *A. annua* and *A. vulgaris*, chlorogenic acid was identified as the most prevalent polyphenol, with luteolin being the second most abundant (Figure 3). Considering that numerous studies have demonstrated significant differences in the chemical composition and biological effects of Artemisia spp. based on the geographical origin of the plants, the specific plant part used for extraction, and the extraction method itself [11,29,30], our findings that chlorogenic acid is a prevalent phenolic compound in *A. annua* and *A. vulgaris* are consistent with other data from the same geographical region [31,32].

Excipients can help to improve extraction yields and the preservation of the structural integrity of sensitive polyphenolic molecules, acting as solubilizers, stabilizers, or modifiers of the extraction environment [26,27]. In our study, the use of excipients such as titanium dioxide (2%) and L-glutathione (1%) significantly improved polyphenol concentration in hydroalcoholic herbal extracts; however, the addition of propylene glycol (10%) was less effective, whereas β-cyclodextrin (5%) in most cases was not effective at all (Table 1). 

Propylene glycol, while beneficial for enhancing the solubility and stability of phenolic compounds, may not always be the optimal excipient for their extraction due to several drawbacks [33]. Its selective solubility can limit the extraction of certain phenolics, and safety concerns arise from its potential toxicity at high concentrations or prolonged intake [33]. Additionally, propylene glycol might alter the natural properties and effectiveness of phenolic compounds [33]. Thus, alternatives could be more favorable for broad-spectrum phenolic extraction, especially in large-scale applications where efficiency, safety, and sustainability are taken into account. Cyclodextrins can form complexes with various compounds through non-covalent forces, including van der Waals forces, hydrophobic interactions, and hydrogen bonds [34]. However, β-cyclodextrin, despite its unique ability to enhance the solubility and stability of hydrophobic molecules through the formation of inclusion complexes, may not be the ideal excipient for the hydroalcoholic extraction of phenolic compounds due to several limitations. Its restricted solubility in water and hydroalcoholic mixtures, selective complexation based on cavity size, and potential alteration of natural properties of phenolic compounds [34] can limit its effectiveness and impact on the desired outcomes of the extraction. Our results also have shown (Table 1) that the yield of the phenolic compounds was not increased, and even had a tendency to be lower when β-cyclodextrin (5%) was used as an excipient during the hydroalcoholic extraction of polyphenols from *A. annua* and *A. vulgaris*. Other studies have shown that the efficiency of β-cyclodextrin in extracting a broad spectrum of phenolic compounds may not be equal to that of more traditional solvents [35,36]. Hydroalcoholic solutions, without the addition of cyclodextrins, can often extract a wider range of phenolics more efficiently due to their ability to dissolve both hydrophilic and hydrophobic compounds [35,36]. The use of titanium dioxide as an excipient in food and pharmaceutical products has been subject recently to regulatory scrutiny due to concerns over its safety, especially in nanoparticle form [28]. Given these considerations, the application of titanium dioxide as an excipient for polyphenolic compounds must be approached with caution. While there are potential benefits in terms of protecting and stabilizing these compounds, the safety and regulatory implications cannot be overlooked.

L-glutathione can protect polyphenolic compounds from oxidative stress during extraction processes due to its antioxidant properties. Moreover, L-glutathione can prevent the polymerization of polyphenolic compounds, a common issue that occurs under oxidative conditions and can negatively affect the efficiency and purity of the extraction [37]. The thiol group of L-glutathione has the potential to interact with specific polyphenolic compounds, thus enhancing the solubility of polyphenols in the extraction solvent [37]. The extraction conditions, including pH, temperature, and duration, as well as the choice of the extraction solvent, need careful monitoring to maintain L-glutathione in its reduced and active state, as its antioxidant properties are dependent on its reduction potential [37]. Nevertheless, incorporating L-glutathione as an excipient in the extraction of polyphenolic compounds could be a novel method to improve the yield, purity, and stability of these bioactive molecules. In our study, the total phenolic content in the extracts of *A. annua* and *A. vulgaris* was enhanced with the addition of 1% L-glutathione as an excipient, compared to the control samples (Figure 4). Additionally, the extracts from *A. vulgaris* had a higher concentration of phenolic compounds than those from *A. annua* (Figure 4), implying that *A. vulgaris* could be a more promising source in the search of novel bioactive substances.

### 3.2. Molecular Docking of Main Bioactive Phenolic Compounds from Herbal Hydroalcoholic Extracts of Artemisia annua L. and Artemisia vulgaris L. with Proteins from Apoptosis and Necroptosis Pathways

Phenolic compounds exhibit a wide range of biological effects by interacting with multiple targets, rather than acting on a specific site [38,39,40]. Both *A. annua* and *A. vulgaris* contain polyphenolic compounds with potential anticancer activities [9,13,41]. These activities are attributed to their ability to modulate various molecular pathways involved in cancer development and progression [9,13,39,40].

In molecular modeling, protein–ligand docking is used to predict the preferred orientation of a ligand when it binds to a protein target at the molecular level. Two key factors in evaluating the strength and specificity of these interactions are the binding energy and the number of hydrogen bonds formed between the protein and ligand [42,43].

We conducted molecular docking studies to explore the interactions between the main phenolic compounds of *A. annua* and *A. vulgaris* and key target proteins involved in apoptotic and necroptosis pathways. Apoptosis is crucial for eliminating damaged, dysfunctional, or potentially cancerous cells, and its dysregulation is a hallmark of many cancers [44,45]. Necroptosis, on the other hand, is a form of programmed cell death that mimics necrosis, traditionally considered a form of accidental cell death caused by external factors. Unlike apoptosis, necroptosis results in cell lysis, releasing cellular contents that can trigger inflammation, making it a double-edged sword in cancer. This pathway is mediated by specific signaling proteins, such as receptor-interacting protein kinases (RIPK1 and RIPK3) and the mixed lineage kinase domain-like pseudokinase (MLKL) [44,46,47]. Both pathways are targets for anticancer therapies due to their ability to control cell survival and death [45]. The modulation of these pathways can help in selectively inducing death in cancer cells, potentially overcoming resistance to conventional therapies [44,45].

In our study, we examined proapoptotic Bax (PDB ID 6EB6), anti-apoptotic Bcl-2 (PDB ID G5M), and the necroptosis pathway protein, mixed lineage kinase domain-like protein (MLKL), in complex with receptor-interacting serine/threonine-protein kinase 3 (RIPK3), indicated by PDB ID 7MON. The results revealed a high probability of interaction (Table 2), especially noting the potential for multiple hydrogen bonds between chlorogenic acid—a primary polyphenol in the Artemisia species—and the MLKL/RIPK3 protein complex (Figure 5), indicating the high probability of the possible interaction between them, and the possible role of chlorogenic acid in the modulation of the processes of necroptosis. However, the molecular docking predictions should be interpreted with caution and ideally confirmed by experimental data.

Recent investigations revealed that chlorogenic acid, in addition to its antioxidant and anti-inflammatory properties, can influence several key molecular pathways involved in cell survival and death [41,48]. In the context of necroptosis, chlorogenic acid may interact with key proteins such as RIPK1, RIPK3, and MLKL, which are central to necroptosis [41,48]. These interactions could potentially modulate the pathway, either inhibiting or facilitating cell death depending on the cellular context and the presence of other signals [41,48]. The ability of chlorogenic acid to cross the blood–brain barrier also makes it a compound of interest in neurodegenerative diseases and brain tumors, where necroptosis might play a critical role [20]. It may have a substantial effect on glioblastoma cells, providing a novel therapeutic avenue in cases where conventional apoptosis-inducing therapies have proven ineffective.

### 3.3. Effects of Artemisia annua L. and Artemisia vulgaris L. Extracts and Chlorogenic Acid on the Viability of the Rat C6 Glioma Cells

The challenge to control glioblastoma due to its aggressive nature and resistance to standard treatments necessitates ongoing research and clinical trials to explore and develop novel techniques. Integrating these new methods into conventional healthcare is crucial for improving patient outcomes and finding more effective therapies [49].

In our study, we evaluated the cell viability of the rat C6 glioma cell line using a nuclear fluorescent double-staining assay with Hoechst 33342 and propidium iodide. The C6 glioma cell line is frequently employed as a model for glioblastoma because it closely mimics human glioma characteristics, including rapid growth and high invasiveness [50]. The results showed that the extract of *A. annua*, at a concentration of 50 µg/mL of phenolic compounds (Figure 6 and Figure 9), and the extract of *A. vulgaris*, at 70 µg/mL (Figure 7), successfully induced necrotic cell death in all tested glioma C6 cells. Moreover, chlorogenic acid, at a concentration of 70 µg/mL, decreased the viability of the rat glioma C6 cells by 48–52% (Figure 8).

In other studies, chlorogenic acid, the most potent functional inhibitor of the microsomal glucose-6-phosphate translocase, could regulate the invasive phenotype of brain tumor-derived primary glioma cells [51]. Chlorogenic acid has been shown to readily cross the blood–brain barrier [52,53,54] and was safe at high doses in mice up to 1000 mg/kg [52]. Pharmacokinetic studies have shown that chlorogenic acid rapidly enters the plasma and reaches the brain (measured concentrations of 250 µg/mL shortly after administration and 25 µg/mL six hours later) and cerebrospinal fluid when administered intranasally and intravenously at doses of 10 mg/kg [53]. These results indicate that concentrations of chlorogenic acid capable of inhibiting cancer could be readily attained in vivo, suggesting its potential usefulness in treating glioblastoma.

Also, several studies have shown the anticancer activity of phenolic plant extracts, containing chlorogenic acid as one of the principal compounds. Silver nanoparticles with the aqueous extract obtained from the green leaves of the *Diospyros kaki* L. (Persimmon), rich in chlorogenic acid (11,210.6471 μg/mL), cynarin, hyperoside, quercetin-3-glucoside, and quercetin-3-D-xyloside were extremely cytotoxic to the CaCo-2 cell line at concentration of 50 µg/mL and had a suppressive effect on the proliferation of glioblastoma U118, Caco-2, and Skov-3 cells [55]. Methanolic fraction of *Cassia fistula* L. bark, rich in phenolic (130.37 mg gallic acid equivalent/g dry weight of extract) and flavonoid (36.96 mg rutin equivalent/g dry weight of fraction) compounds: catechin, gallic acid, chlorogenic acid, and kaempferol, reduced over 50% cell growth at the concentration of 76.72 µg/mL in A431 cells [56]. Japanese quince (*Chaenomeles japonica* (Thunb. Lindl. ex Spach) leaf ethanol extracts, rich in phenolic compounds, could efficiently reduce (EC_50_ 1.26 mg/mL) glioblastoma HROG36 cell viability while preserving non-cancerous cells [57]. *Leonurus sibiricus* L. non-transformed and transformed root extracts rich in phenolic acids (8.14 and 17.44 mg/g dry weight, respectively); chlorogenic, caffeic, and ellagic acid as the main constituents, were found to have cytotoxic activity (EC_50_ 2.4 mg/mL) on the primary glioma cells [58].

In our study, *A. annua* (Figure 6) and *A. vulgaris* (Figure 7) extracts were more potent than chlorogenic acid (Figure 8). Whole extracts rich in polyphenolic compounds often exhibit higher anticancer activity compared to single compounds due to a phenomenon known as the synergistic effect [59,60,61,62]. This effect occurs when multiple compounds within the extract interact to enhance each other’s therapeutic properties, leading to more potent biological effects [59,60,61,62]. Different polyphenols may target multiple pathways involved in cancer progression, such as inhibiting tumor growth, inducing apoptosis in cancerous cells, and preventing angiogenesis [59,61]. Additionally, some polyphenols can enhance the bioavailability or stability of others, increasing their overall efficacy [59,61]. This synergistic interaction can reduce the likelihood of cancer cells developing resistance to treatment, which is a common issue with therapies based on single compounds [59,61]. Thus, using whole extracts containing a diverse array of polyphenolic compounds can offer a broader and more effective approach to cancer prevention and treatment.

## 4. Materials and Methods

### 4.1. Plant Materials, Chemicals, and Equipment

The dried herbs of *A. annua* L. and *Artemisia vulgaris* L. were purchased from UAB “Jadvygos žolės” (Mazeikiu raj, Lithuania). The chemicals used for this study were 96% ethanol (Vilnius, Lithuania), L-Glutathione ≥98% (ROTH,, Karlsruhe, Germany), propylene glycol, titanium dioxide SOLAVEIL XT-40W-LQ-(WD), and β-Cyclodextrin (Sigma-Aldrich, Schnelldorf, Germany).

The dried herbs of *A. annua* and *A. vulgaris* were milled to powder-like substances using a trapezoid hole sieve with the size of 0.5 mm at 8000 rpm speed using the Ultra Centrifugal Mill ZM 200 (Retsch, Haan, Germany). The equipment used for the extractions were the ultrasound waterbath “Grant XUB10” (Grant Instruments, Cambridge, UK) and the centrifuge “Sigma 3-18KS” (SIGMA Laborzentrifugen, Osterode am Harz, Germany).

### 4.2. Preparation of Artemisia Plant Extracts

Various hydroalcoholic extracts of Artemisia spp. were prepared at concentrations of 100, 300, and 500 mg/mL. The milled plant material was mixed with different types of ethanol concentrations. The concentrations were used as follows: 50%, 60%, 70%, 80%, and 96% (*w*/*w*). The samples were sonicated at 200 W, with the frequency of 38 kHz for 30 min at 25 °C. Afterwards, the samples were centrifuged for 15 min at 3000 rpm speed and finally filtered with filter paper. After the high-performance liquid chromatography analysis was performed on the samples, one satisfactory ethanol concentration was selected for further extract preparation. The extracts were enriched with the selected excipients: titanium dioxide (2%), L-glutathione (1%), propylene glycol (10%), and β-cyclodextrin (5%). The extractions were obtained under identical conditions using ultrasound-assisted extraction (UAE). The extractions were analyzed with high-performance liquid chromatography (HPLC).

### 4.3. High-Performance Liquid Chromatography with Diode Array Detector (HPLC-DAD) Conditions for the Extract Analysis

The predominant phenolic compounds in Artemisia spp. extracts were detected using high-performance liquid chromatography (HPLC), using a Waters e2695 chromatographic system with an ACE 5 C18 chromatography column (250 × 4.6 mm) and a Waters 2998 diode array detector [63]. The obtained data were processed by the Waters^®^ Empower^®^ 3 Chromatography Data System. HPLC eluents consisted of 0.1% trifluoroacetic acid (eluent A) and 100% acetonitrile (eluent B). The elution program was used as follows: from 5% to 15% eluent B at 0–8 min, from 15% to 20% eluent B at 8–30 min, from 20% to 40% eluent B at 30–48 min, from 40% to 50% eluent B at 48–58 min, from 50% to 50% eluent B at 58–65 min, from 50% to 95% eluent B at 65–66 min, from 95% to 95% eluent B at 66–70 min, and from 95% to 5% eluent B at 70–71 min. The mobile phase flow rate was 1 mL/min. The injection volume of extract was 10 µL. The column temperature was 25 °C. Compounds present in the samples were identified by the UV absorption at a wavelength range of 300–400 nm and by the retention time of analytes and reference substances.

### 4.4. Molecular Docking

The structures of studied compounds were obtained from the PubChem database (https://pubchem.ncbi.nlm.nih.gov/ (accessed on 20 May 2024)). Molecules were converted into PDBQT format to perform docking studies. Crystal structures of target proteins were retrieved from the Protein Data Bank (RCSB PDB, http://www.rcsb.org/ (accessed on 20 May 2024)): the pro-apoptotic protein Bax (PDBID: 6EB6), anti-apoptotic protein Bcl-2 (PDBID: 1G5M), and necroptosis pathway mixed lineage kinase domain-like protein (MLKL) in complex with receptor-interacting serine/threonine-protein kinase 3 (RIPK3) (PDB ID: 7MON). All non-protein residues were removed, retaining a pure protein structure for docking simulations. Molecular docking studies were carried out using AutoDock Vina 4.05 [64]. Energy was minimized with universal force field using a conjugate gradient algorithm with 200 run iterations. The docking was accomplished using a Lamarckian genetic algorithm with local search. Docking parameters were set as follows: the number of individuals in the population was 150, the maximum number of energy evaluations was 25,000, the maximum number of generations was 30,000, the top individual to survive to the next generation was 1, the gene mutation rate was 0.02, the crossover rate was 0.8, the Cauchy beta was 1.0, and the genetic algorithm window size was 10.0. The box space covered all the interior of the protein studied and the protein itself. Structures with the lowest docking energy, that is, the highest affinity to the studied protein, were analyzed using PyMOL v 3.0.

### 4.5. Cell Viability Assessment In Vitro

All cell culture reagents were obtained from Gibco (Fisher Scientific, Loughborough, UK). Rat glioma C6 cells were purchased from the Cell Lines Service GmbH, Eppelheim, Germany. For the cytotoxicity control of tested extracts or chlorogenic acid, a rat cerebellar neuronal-glial cell culture was used from postnatal 5–7-day-old Wistar rats [65,66]. Cell cultures were seeded in culture flasks containing Dulbecco’s Modified Eagle Medium (DMEM) with 10% of fetal bovine serum, 100 U/mL penicillin, and 100 µg/mL streptomycin. The cultures were then incubated at 37 °C, with 5% CO_2_ and saturated humidity. Twenty-four h prior to treatment with investigated extracts or chlorogenic acid, the cells were transferred to 96-well plates at a density of 20,000 cells/well. In the first experimental series, cell cultures were treated with different concentrations (5–70 µg/mL) of extracts or chlorogenic acid for 24 h. After incubation with investigated solutions, the cells were double-stained with Hoechst 33342 (15 µg/mL) and propidium iodide (PI; 5 µg/mL) for 15 min, and the viability was assessed under fluorescence microscope OLYMPUS IX71SIF-3 (Olympus Optical Co., Ltd., San Jose, CA, USA). Necrotic versus viable cells were identified using the DAPI filter set with an excitation band pass of 352–402 nm and a long pass emission filter transmitting light waves longer than 410 nm [66]. Hoechst33342-only-positive nuclei exhibiting blue fluorescence were considered viable, and Hoechst3334-plus-PI-positive nuclei fluorescing magenta were identified as necrotic. Small nuclei with condensed chromatin, visible as very bright blue, were considered apoptotic [66].

### 4.6. Statistical Analysis

Data are presented as mean ± SEM, as indicated. Statistical analysis was performed using a one-way analysis of variance (ANOVA), followed by Tukey’s multiple comparison test or Dunnett’s post hoc test, using the software package Prism v. 10.12 (GraphPad Software Inc., La Jolla, CA, USA) or the software package SigmaPlot v. 13.0 (Grafiti LLC, Palo Alto, CA, USA). A value of *p* < 0.05 was taken as the level of significance.

## 5. Conclusions

In conclusion, our findings indicate that the extracts from *A. annua* and *A. vulgaris* exhibit potent anticancer activity in a glioblastoma model. The synergistic effects of their combined compounds were more effective than any single compound alone, underscoring the potential of these extracts as a foundation for developing more effective glioblastoma treatments. Further investigations of these plants and their polyphenolic compounds could lead to the development of new anticancer drugs or adjunct therapies to enhance the efficacy of existing treatments. While there is promising preliminary evidence, further research, including clinical trials, is necessary to fully understand the potential of these compounds in cancer therapy, their efficacy, safety, and mechanisms of action.

## Figures and Tables

**Table 1:** Effects of the excipients added to the extraction mixture on the yield of polyphenolic compounds in different hydroalcoholic extracts of *Artemisia annua* L. and *Artemisia vulgaris* L. herbs.

Concentration, µg/mL	Control	Titanium Dioxide 2%	L-Glutathione 1%	Propylene Glycol 10%	β-Cyclodextrin 5%
***Artemisia annua***					
Chlorogenic acid	582.0 ± 30.2	664.5 ± 28.0 *	643.9 ± 34.7 *	596.3 ± 29.1	498.5 ± 25.6
Luteolin	387.8 ± 18.3	443.9 ± 24.1 *	623.1 ± 35.4 *	556.7 ± 24.2 *	500.1 ± 100.4
Isoquercitrin	48.64 ± 4.20	59.49 ± 5.53 *	61.10 ± 3.20 *	55.84 ± 5.65	47.87 ± 4.54
Apigenin	2.25 ± 0.10	2.75 ± 0.16 *	3.63 ± 0.18 *	3.25 ± 0.17 *	2.67 ± 0.46
Neochlorogenic acid	20.81 ± 1.65	25.14 ± 1.32 *	24.73 ± 1.32 *	22.31 ± 1.31	18.59 ± 1.74
4-o-Caffeoyl-quinic acid	9.23 ± 0.24	10.11 ± 0.35 *	9.68 ± 0.15 *	9.00 ± 0.96	7.62 ± 0.26
Caffeic acid	15.50 ± 2.42	14.49 ± 2.25	15.15 ± 2.65	17.34 ± 3.20	15.50 ± 2.43
***Artemisia vulgaris***					
Chlorogenic acid	1233.7 ± 20.1	1432.6 ± 27.5 *	1293.9 ± 28.9 *	1351.9 ± 52.7 *	1320.2 ± 83.6
Luteolin	265.1 ± 13.2	691.8 ± 23.4 *	387.0 ± 19.2 *	581.3 ± 20.8 *	439.3 ± 26.5 *
Isoquercitrin	1.46 ± 0.12	1.76 ± 0.14 *	1.85 ± 0.17 *	3.26 ± 0.35 *	2.50 ± 0.25 *
Apigenin	1.59 ± 0.17	3.61 ± 0.42 *	2.53 ± 0.19 *	2.54 ± 0.21 *	2.18 ± 0.58
Neochlorogenic acid	24.00 ± 1.60	35.26 ± 5.83 *	29.39 ± 1.71 *	29.82 ± 4.95	26.32 ± 3.54
4-o-Caffeoyl-quinic acid	22.67 ± 2.25	33.16 ± 4.87 *	27.90 ± 2.27 *	27.71 ± 4.78	27.20 ± 5.1
Caffeic acid	12.06 ± 0.35	15.42 ± 1.72 *	13.03 ± 0.23 *	14.14 ± 1.21 *	12.32 ± 1.87

Data are presented as mean ± SEM, n = 4. * *p* < 0.05—statistically significant effect of corresponding excipient compared to control without excipients. The results were analyzed with a one-way analysis of variance (ANOVA) followed by Dunnett’s post hoc test.

**Table 2:** Binding affinities and the number of formed hydrogen bonds of main polyphenolic compounds of *Artemisia annua* L. and *Artemisia vulgaris* L. docked to selected proteins from apoptosis and necroptosis pathways.

	Bax	Bcl-2	MLKL/RIPK3
Chlorogenic acid	−7.4 kcal/mol N6	−7.4 kcal/mol N9	−6.8 kcal/mol N18
Luteolin	−6.6 kcal/mol N6	−8.1 kcal/mol N9	−8.5 kcal/mol N5
Isoquercitrin	−7.6 kcal/mol N10	−8.3 kcal/mol N7	−9.2 kcal/mol N11
Apigenin	−6.3 kcal/mol N5	−7.8 kcal/mol N6	−7.2 kcal/mol N4
Neochlorogenic acid	−6.9 kcal/mol N7	−7.6 kcal/mol N8	−6.9 kcal/mol N10
4-o-Caffeoylquinic acid	−7.0 kcal/mol N10	−7.7 kcal/mol N5	−7.2 kcal/mol N5
Caffeic acid	−5.2 kcal/mol N4	−6.1 kcal/mol N6	−6.3 kcal/mol N10

## Data Availability

Data are contained within the article.
